# MAL-Net: A Multi-Label Deep Learning Framework Integrating LSTM and Multi-Head Attention for Enhanced Classification of IgA Nephropathy Subtypes Using Clinical Sensor Data

**DOI:** 10.3390/s25061916

**Published:** 2025-03-19

**Authors:** Hongyan Wang, Yuehui Liao, Li Gao, Panfei Li, Junwei Huang, Peng Xu, Bin Fu, Qin Zhu, Xiaobo Lai

**Affiliations:** 1 School of Medical Technology and Information Engineering, Zhejiang Chinese Medical University, Hangzhou 310053, China; whyan@zcmu.edu.cn (H.W.); zcmlyh@zcmu.edu.cn (Y.L.); lipanfei@zcmu.edu.cn (P.L.); geli@zcmu.edu.cn (J.H.); 2 Hangzhou TCM Hospital Affiliated to Zhejiang Chinese Medical University, Hangzhou 310005, China; gaoligaoli2024@163.com; 3 Digital Chinese Medicine Institute, Zhejiang Chinese Medical University, Hangzhou 310053, China; fubin@zcmu.edu.cn; 4 Third Affiliated Hospital, Zhejiang Chinese Medical University, Hangzhou 310005, China; 600xup@163.com

**Keywords:** IgA nephropathy (IgAN), subtype classification, long short-term memory (LSTM), attention mechanism, multi-label classification, clinical sensors

## Abstract

Background: IgA nephropathy (IgAN) is a leading cause of renal failure, characterized by significant clinical and pathological heterogeneity. Accurate subtype classification remains challenging due to overlapping clinical manifestations and the multidimensional nature of data. Traditional methods often fail to fully capture IgAN’s complexity, limiting their clinical applicability. This study introduces MAL-Net, a deep learning framework for multi-label classification of IgAN subtypes, leveraging multidimensional clinical data and incorporating sensor-based inputs such as laboratory indices and symptom tracking. Methods: MAL-Net integrates Long Short-Term Memory (LSTM) networks with Multi-Head Attention (MHA) mechanisms to effectively capture sequential and contextual dependencies in clinical data. A memory network module extracts features from clinical sensors and records, while the MHA module emphasizes critical features and mitigates class imbalance. The model was trained and validated on clinical data from 500 IgAN patients, incorporating demographic, laboratory, and symptomatic variables. Performance was evaluated against six baseline models, including traditional machine learning and deep learning approaches. Results: MAL-Net outperformed all baseline models, achieving 91% accuracy and an AUC of 0.97. The integration of MHA significantly enhanced classification performance, particularly for underrepresented subtypes. The F1-score for the Ni-du subtype improved by 0.8, demonstrating the model’s ability to address class imbalance and improve precision. Conclusions: MAL-Net provides a robust solution for multi-label IgAN subtype classification, tackling challenges such as data heterogeneity, class imbalance, and feature interdependencies. By integrating clinical sensor data, MAL-Net enhances IgAN subtype prediction, supporting early diagnosis, personalized treatment, and improved prognosis evaluation.

## 1. Introduction

IgA nephropathy (IgAN) is the most prevalent primary glomerular disease worldwide and a major contributor to chronic kidney disease (CKD) and end-stage renal disease (ESRD) [1,2]. It exhibits substantial clinical, pathological, and prognostic heterogeneity. While some patients experience a benign disease course, others progress rapidly to ESRD, requiring renal replacement therapy [3,4]. Accurate diagnosis and timely intervention are critical for managing IgAN; however, subtype classification remains challenging due to overlapping clinical and pathological features.

Current classification systems, including the Lee grading system, Haas classification, and the widely adopted Oxford classification, primarily emphasize histopathological features such as mesangial hypercellularity, endocapillary proliferation, and segmental glomerulosclerosis [5,6,7]. While valuable for assessing disease severity and prognosis, these systems often fail to incorporate essential clinical information, such as patient demographics, laboratory indices, and symptoms. Moreover, these approaches adopt a single-label classification paradigm, assigning each patient to a single category, despite evidence of overlapping subtypes in IgAN [8].

Machine learning (ML) techniques have emerged as powerful tools in medical diagnostics, classification, and disease prognosis, enabling data-driven clinical decision-making [9,10,11,12,13,14]. Traditional ML methods, such as Support Vector Machines (SVM), Random Forest (RF), and Logistic Regression (LR), have been used to predict IgAN occurrence and severity [15,16,17,18,19]. However, these methods struggle with the high-dimensional, heterogeneous, and sequential nature of clinical data. They also rely heavily on manual feature engineering, which limits their ability to capture intricate relationships between features and IgAN subtypes [4]. Additionally, traditional ML methods often underperform in multi-label tasks due to their inability to handle label dependencies and overlapping subtypes.

Deep learning (DL) offers significant advantages over traditional ML by automatically extracting hierarchical and non-linear relationships from complex datasets [20]. For example, Ren [10] developed a hybrid neural network for CKD prediction using electronic health record (EHR) data. Similarly, Ren et al. [16] constructed a single-sample subtype classifier (SSRC) based on the GEO database and multiple unsupervised clustering algorithms, identifying three functional subtypes of IgAN: viral–hormonal, bacterial–immune, and mixed types. Furthermore, Schena et al. [18] used artificial neural networks to classify IgAN severity in a retrospective cohort of over 900 patients. Among DL models, Recurrent Neural Networks (RNNs) and their variants, such as Long Short-Term Memory (LSTM) networks, are particularly effective for processing sequential and temporal data [21]. LSTMs excel in learning long-term dependencies, making them ideal for tracking variations in laboratory indices, symptom progression, and disease dynamics [22,23,24,25,26]. However, LSTM models often struggle to differentiate critical features in heterogeneous data, especially when faced with class imbalance and noisy inputs—common challenges in IgAN subtype classification.

To overcome these limitations, attention mechanisms have been widely adopted to enhance model performance. These mechanisms enable models to focus on the most relevant features, improving their ability to capture complex relationships and reduce the influence of irrelevant or noisy data [27]. The Multi-Head Attention (MHA) mechanism enhances feature selection by focusing on multiple aspects of input data simultaneously, improving the detection of subtle but important patterns within clinical datasets [28]. This makes MHA particularly effective for multi-label classification tasks, where understanding inter-label correlations and feature dependencies is crucial.

Given these advancements and challenges, this study proposes MAL-Net, a novel deep learning framework that integrates LSTM networks and MHA mechanisms for the multi-label classification of IgAN subtypes. MAL-Net leverages multidimensional clinical data—including demographic characteristics, laboratory indices, and symptomatic features—to predict the co-occurrence of multiple IgAN subtypes. The model addresses key challenges such as data heterogeneity, class imbalance, and subtype complexity, providing clinicians with a reliable tool for IgAN subtype classification. This, in turn, supports early diagnosis, personalized treatment planning, and improved prognosis evaluation, ultimately enhancing patient outcomes. The primary contributions of this study are threefold:

(1) Development of MAL-Net: A hybrid deep learning model that combines LSTM and MHA mechanisms to effectively capture sequential relationships and feature dependencies within heterogeneous clinical data.

(2) Rigorous Evaluation: Comprehensive assessment of MAL-Net using multiple performance metrics, including accuracy, precision, recall, F1-score, and area under the receiver operating characteristic curve (AUC), demonstrating its robustness and clinical applicability.

(3) Comparative Analysis: Benchmarking MAL-Net against six baseline models, including traditional ML methods (RF, SVM, LR) and deep learning approaches (DNN, CNN, LSTM), highlighting its superiority in multi-label classification tasks.

## 2. Materials and Methods

### 2.1. Patient Selection and Data Collection

**Figure 1:**
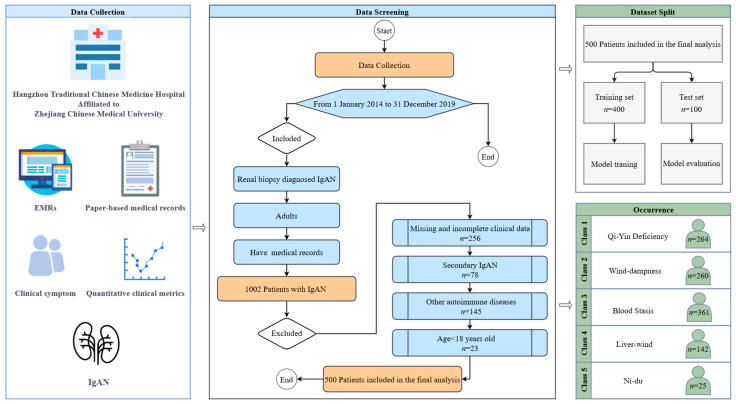
Flow diagram of sample selection.

This study retrospectively analyzed clinical data from 1002 patients diagnosed with IgA nephropathy (IgAN) through renal biopsy at Hangzhou Traditional Chinese Medicine Hospital, Zhejiang Chinese Medical University, between 1 January 2014, and 31 December 2019. Patient data were collected through a combination of manual review of paper-based medical records and automated retrieval from electronic medical records (EMRs). Inclusion criteria: (1) patients diagnosed with IgAN confirmed by renal biopsy; (2) availability of complete clinical data, including demographic, symptomatic, and laboratory indices. Exclusion criteria: (1) patients under the age of 18; (2) patients with incomplete or missing clinical data (patients whose data were missing for more than 20% of any variable were excluded). After applying the inclusion and exclusion criteria, a total of 500 patients were included in the final dataset for model development. The patient selection process is illustrated in Figure 1.

### 2.2. Subtype Classification

Patients were classified into five distinct IgAN subtypes—Qi-Yin Deficiency, Wind-dampness, Liver-wind, Blood Stasis, and Ni-du—according to their clinical features and pathological characteristics. This classification adheres to the diagnostic criteria established by Hangzhou Traditional Chinese Medicine Hospital, affiliated with Zhejiang Chinese Medical University, which integrates traditional clinical manifestations and underlying disease mechanisms. Given that many patients exhibited overlapping characteristics indicative of multiple subtypes, we employed a multi-label classification approach. This allowed the model to accurately represent the clinical complexity and coexistence of different IgAN subtypes within individual patients.

### 2.3. Predictor Variables

To comprehensively capture the clinical characteristics relevant to IgAN subtype classification, patient data included demographic variables (age and sex), laboratory indices (systolic blood pressure [SBP], diastolic blood pressure [DBP], urinary protein content [UPC], dysmorphic red blood cells [DRBCs], and estimated glomerular filtration rate [eGFR]), and symptom presentations (dizziness, hypertension, hematuria, lumbar pain, fatigue, edema, nocturia, and dry throat). The distribution of these features across training and test datasets is summarized in Table 1. Statistical correlation tests indicated no significant differences (all *p*-values > 0.05) between the training and test sets, confirming balanced data partitioning and minimal potential bias, thereby ensuring comparability and robustness in model evaluation.

### 2.4. Data Preprocessing

To ensure high-quality input data and minimize biases, comprehensive data preprocessing was conducted, including the following steps:

#### 2.4.1. Missing Data Handling

A threshold-based exclusion criterion was applied to remove cases with excessive missing clinical data (>20%). For cases with minimal missing data (≤20%), appropriate imputation techniques were used to preserve data integrity. Specifically, missing numerical variables (e.g., age, SBP, DBP) were imputed using mean imputation, while missing categorical variables (e.g., symptom presence) were handled using mode imputation.

#### 2.4.2. Feature Encoding

Categorical features, including symptoms and sex, were transformed into numerical values using one-hot encoding. This approach allowed the model to handle categorical variables in a way that preserves their relationship with the target labels while ensuring compatibility with the deep learning framework.

#### 2.4.3. Feature Normalization

Continuous features such as age, SBP, DBP, and UPC were normalized to improve model training stability and convergence speed. A min-max scaling method was applied to standardize the values of each feature to the range [0, 1]:

(1) $$x_{normalized}=\frac{x-x_{min}}{x_{max}-x_{min}},$$

where x is the original feature value, while $x_{\min}$ and $x_{\max}$ represent the minimum and maximum values of the feature in the training dataset, respectively.

#### 2.4.4. Class Imbalance Mitigation

Given the observed class imbalance in the dataset—particularly the underrepresentation of certain subtypes (e.g., Ni-du)—several techniques were implemented to reduce bias in model predictions. Specifically, the model was trained using a weighted loss function, assigning higher weights to minority subtypes to enhance their representation during training.

#### 2.4.5. Training and Test Sets

The data were randomly split into a training set (80%) and a test set (20%), ensuring a balanced distribution of all IgAN subtypes. This partitioning facilitated unbiased model training and evaluation.

### 2.5. Model Development: MAL-Net Architecture

**Figure 2:**
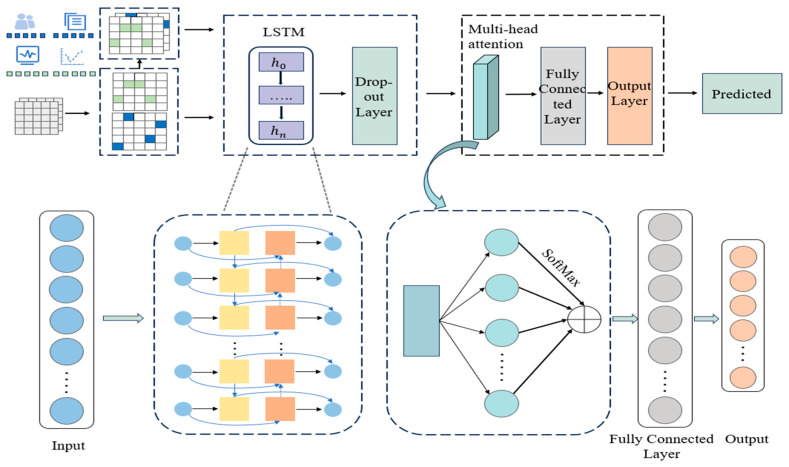
Architecture of the MAL-Net.

The MAL-Net framework integrates LSTM networks and the MHA mechanism to address the challenges of multi-label IgAN subtype classification. As illustrated in Figure 2, the architecture consists of two main components: the Memory Network Module and the Matt Module. The Memory Network Module leverages LSTM networks to extract clinical features and model relationships between them. However, the complexity of multi-label classification, combined with a limited sample size in certain subtypes, makes it difficult to capture these relationships effectively. To address these challenges, the Matt Module incorporates the MHA mechanism, enabling the model to identify latent feature interactions, emphasize critical clinical features, and recognize the primary manifestations of each IgAN subtype, ultimately enhancing classification accuracy.

The process begins with preprocessing textual symptoms and numerical indicators, followed by deep feature extraction using the Memory Network Module. To prevent overfitting, regularization is applied via a dropout layer. The extracted feature representations are then processed by the Matt Module, where the Multi-Head Attention (MHA) mechanism assigns adaptive weights to different input features. These weighted representations are then aggregated to enhance feature expressiveness.

Next, the enhanced features are passed through a fully connected layer for further feature extraction and nonlinear transformations. Finally, the output layer generates independent probability predictions for each subtype. Subtypes with prediction probabilities exceeding a predefined threshold are classified as positive, enabling multi-label classification of patients.

The model parameters are updated via backpropagation using the Adam optimization algorithm, which adaptively adjusts learning rates based on first- and second-moment gradient estimates [29]. This dynamic learning rate adjustment enhances training efficiency, improves convergence speed, and optimizes model performance.

#### 2.5.1. Memory Network Module (LSTM Layer)

The Memory Network Module is built upon the LSTM network architecture [30], leveraging its strengths in sequential learning and feature extraction. By incorporating enhancements, the LSTM layer improves memory capacity, enabling the precise extraction of multi-dimensional input features. This architecture captures contextual information, models complex nonlinear relationships, and preserves the sequential structure of textual data. These capabilities enhance the processing of sequential information and provide deeper insights into clinical symptoms and their temporal variations in disease progression.

(1) Gating mechanism structure of LSTM unit

**Figure 3:**
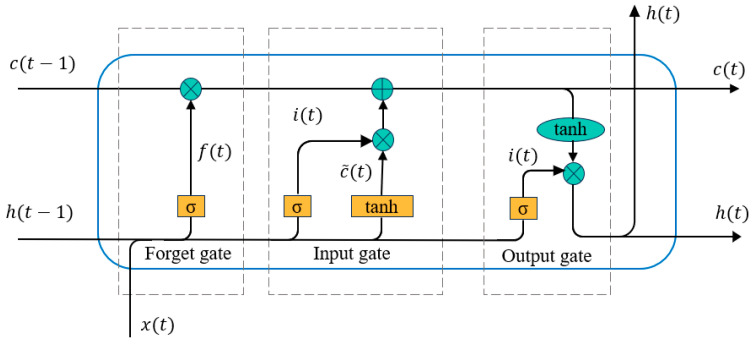
Gating mechanism structure of LSTM unit.

As illustrated in Figure 3, the LSTM unit comprises three gating mechanisms-input gate, forget gate, and output gate—along with a cell state. The cell state stores long-term information, while the gates regulate the flow and retention of information as follows:

Input Gate ($i_{t}$): Determines which information enters the cell state, based on the preprocessed features.

Forget Gate ($f_{t}$): Decides which information to discard.

Output Gate ($o_{t}$): Controls which information is passed out from the cell.

Through these gates, new hidden states $\left(h_{t}\right)$ and cell states $\left(c_{t}\right)$ are generated during each time step of the sequence. The mathematical operations are defined as:

(2) $$i_{t}=\sigma \left(W_{i}x_{t}+U_{i}h_{t-1}+b_{i}\right),$$

(3) $$\begin{array}{c} f_{t}=\sigma \left(W_{f}x_{t}+U_{f}h_{t-1}+b_{f}\right) \end{array},$$

(4) $${\tilde{c}}_{t}=\tanh\left(W_{c}x_{t}+U_{c}h_{t-1}+b_{c}\right),$$

(5) $$c_{t}=ft⊙c_{t-1}+i_{t}⊙{\tilde{c}}_{t},$$

(6) $$h_{t}=o_{t}⊙\tanh\left(c_{t}\right),$$

where $x_{t}$ represents the input at time t, $h_{t-1}$ is the previous hidden state, W and U are weight matrices, b is the bias term, σ denotes the sigmoid activation function, tanh represents the hyperbolic tangent activation function, ⊙ is the element-wise multiplication operator. This gating mechanism ensures the effective integration of long-term dependencies and real-time updates for the input sequence.

(2) Dropout layer

To further enhance the generalization ability of the model and mitigate overfitting, a Dropout layer is applied after the LSTM layer [31]. While the LSTM layer captures long-term dependencies effectively, overfitting risks arise during training, leading to suboptimal performance on unseen data. The Dropout layer addresses this by randomly deactivating a proportion of neurons during each training iteration, with a specified probability (P). This introduces sparsity, preventing the model from over-relying on specific features or paths and fostering more robust and generalized feature learning.

The processed output from the LSTM layer is passed through the dropout layer, where a new hidden state ($h_{t}^{\prime }$) is generated as:

(7) $$\begin{array}{c} h_{t}^{\prime }=\mathrm{Dropout}\left(h_{t}\right) \end{array},$$

where certain neurons are randomly dropped based on the probability P.

By discouraging reliance on specific neural paths or feature combinations, this mechanism reduces overfitting, enhances training stability, and improves the model’s predictive ability on unseen data. The combination of LSTM and Dropout layers ensures robust feature extraction and generalization, critical for the multi-label classification task.

#### 2.5.2. MHA

The MHA mechanism addresses class imbalance by improving feature learning in underrepresented categories, which pose challenges for the Memory Network Module. To mitigate these limitations, the Matt Module integrates MHA, leveraging the attention mechanism’s ability to mimic the brain’s selective focus on relevant signals while filtering out noise. By employing parallel distributed computation, MHA enhances feature selection, reduces interference, and improves information processing efficiency.

(1) Structure of multi-head attention

**Figure 4:**
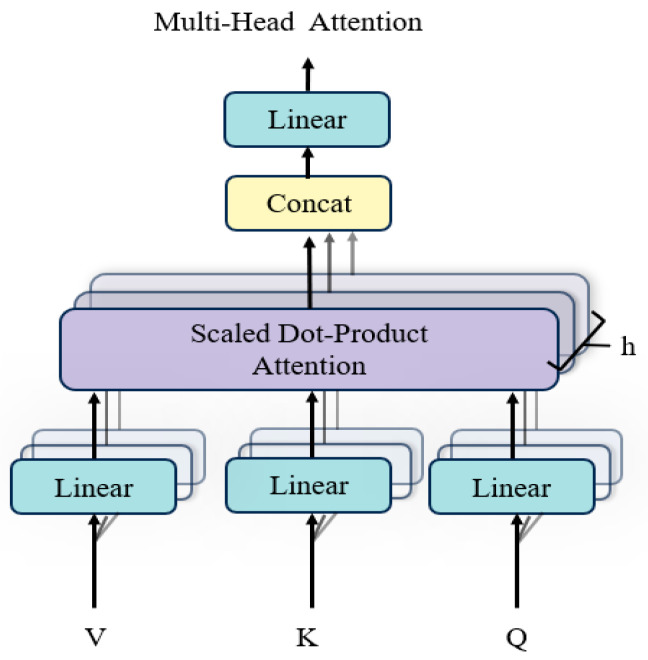
Structure of multi-head attention.

The structure of the MHA layer is depicted in Figure 4. Each attention head independently computes attention scores based on queries (Q), keys (K), and values (V). The attention mechanism operates as follows:

The attention mechanism operates through a series of well-defined steps to compute the final output. First, the similarity between the Q and K vectors is computed using a dot product, resulting in an attention weight matrix. This matrix is then scaled by dividing each value by the square root of the dimensionality of the key vectors ($\sqrt{d_{k}}$), a process designed to mitigate the risk of excessively large values and enhance numerical stability. Next, the scaled values are passed through the SoftMax function to normalize them into a probability distribution, which represents the attention weights associated with the Q in relation to the K. Finally, the attention weight matrix is multiplied by the V vectors to produce the attention output, capturing the most relevant information for subsequent processing. The attention computation is summarized as:

(8) $$\mathrm{Attention}\left(Q,K,V\right)=\mathrm{SoftMax}\left(\frac{QK^{T}}{\sqrt{d_{k}}}\right)V,$$

(2) Parallel attention heads

The MHA mechanism uses multiple parallel attention heads, each independently projecting Q, K, and V into different subspaces using learnable weight matrices $W_{Q}^{\left(h\right)}$, $W_{K}^{\left(h\right)}$, and $W_{V}^{\left(h\right)}$. For each attention head h, the output is computed as:

(9) $${\mathrm{head}}_{h}=\mathrm{Attention}\left(QW_{Q}^{\left(h\right)},KW_{K}^{\left(h\right)},VW_{V}^{\left(h\right)}\right),$$

The outputs of all attention heads are concatenated and transformed using a final projection matrix $W_{O}$:

(10) $$\mathrm{MHA}\left(Q,K,V\right)=\mathrm{Concat}\left({\mathrm{head}}_{1},\ldots {,\mathrm{head}}_{H}\right)W_{O},$$

where H is the number of attention heads, $W_{O}$ is the output projection matrix.

(3) Feature extraction and diversity

The MHA mechanism plays a pivotal role in improving the performance of the multi-label IgAN subtype classification model by leveraging multiple attention heads to capture diverse features and relationships between different subtypes. Each attention head works in parallel, attending to various aspects of the input data, which helps the model overcome challenges like data heterogeneity and class imbalance. This approach allows the model to focus on crucial features while disregarding irrelevant ones, leading to a more comprehensive understanding of the data. By distributing the computation across several heads, the MHA mechanism significantly improves the model’s ability to capture intricate relationships between features, resulting in better feature diversity and more accurate classification outcomes. The integration of this attention mechanism ensures the model can handle complex, multi-dimensional data effectively, which is crucial for tasks like IgAN subtype classification.

#### 2.5.3. Output Layer

After the attention mechanism, the output features are passed through a fully connected layer to generate probabilities for each of the IgAN subtypes. A Sigmoid activation function is applied to output a probability for each label:

(11) $$P\left(y_{i}\right)=\sigma \left(z_{i}\right)=\frac{1}{1+e^{-z_{i}}},$$

where $P\left(y_{i}\right)$ is the probability for the i-th subtype, and $z_{i}$ is the output logits.

#### 2.5.4. Loss Function

The model was trained using the BCEWithLogitsLoss function, which combines the sigmoid activation function and binary cross-entropy loss into a single, efficient operation. This approach allows direct handling of logits, simplifying the training process. Specifically, it is well suited for multi-label classification tasks, as it treats each subtype prediction independently as a binary classification problem. The loss function is mathematically expressed as follows:

(12) $$\begin{array}{c} \mathrm{Loss}=-\frac{1}{N}\overset{N}{\underset{i=1}{\sum }}[w_{i}\cdot y_{i}\cdot \log\left(\sigma \left({\overset{⌢}{y}}_{i}\right)\right) \end{array}+\left(1-y_{i}\right)\cdot \log\left(1-\sigma \left({\overset{⌢}{y}}_{i}\right)\right)],$$

where $\sigma \left({\overset{⌢}{y}}_{i}\right)$ is the Sigmoid activation applied to the raw logits $\left({\overset{⌢}{y}}_{i}\right)$, $y_{i}$ is the true label for sample i, and N is the total number of samples. $w_{i}$ is the weight parameter used to handle class-imbalance data, ensuring that underrepresented subtypes receive sufficient attention, reducing bias towards the majority class. The weight parameter is expressed as:

(13) $$w_{i}=\frac{N_{negative,i}}{N_{positive,i}},$$

where $N_{negative,i}$ and $N_{positive,i}$ are the number of negative and positive samples for subtype i, respectively.

## 3. Results

### 3.1. Experimental Setting

#### 3.1.1. Implementation Details

We used PyCharm as the integrated development environment (IDE) and PyTorch 2.1.0 as the deep learning framework to develop and train the proposed MAL-Net classification model. The MAL-Net framework was implemented with optimized hyperparameters for multi-label IgAN subtype classification. The hidden layer contains 32 nodes, and the Matt Module employs four attention heads. The dataset was split 80:20 into training and testing subsets, ensuring a balanced evaluation. The fully connected layer in the Matt Module utilizes the ReLU activation function to introduce non-linearity. To address class imbalance, the model applies BCEWithLogitsLoss, which integrates binary cross-entropy with the sigmoid activation function, ensuring stable optimization. The model was trained for 500 epochs with an initial learning rate of 0.001, using the Adam optimizer, which dynamically adjusts learning rates based on moment estimates of gradients. A dropout rate of 0.2 was applied to prevent overfitting.

For baseline comparisons, several machine learning and deep learning models were evaluated. The RF model consists of 100 decision trees using Gini impurity as the splitting criterion. The SVM employs a radial basis function (RBF) kernel, with optimal hyperparameters selected via grid search with cross-validation. The LR model applies L2 regularization to mitigate overfitting. The CNN includes two convolutional layers with ReLU activation, while the DNN comprises three hidden layers (128, 64, and 32 neurons, respectively), incorporating batch normalization and dropout regularization to enhance generalization. All neural network models were trained under identical data conditions using the Adam optimizer.

To ensure robustness and fairness, all experiments were evaluated using five-fold cross-validation. This approach minimizes bias and improves model reliability, ensuring that the MAL-Net framework performs effectively across different subsets of the dataset.

#### 3.1.2. Evaluation Metrics

To assess the model’s performance, five widely used evaluation metrics are employed: accuracy, precision, recall, F1-score, and AUC. These metrics are derived from the Confusion Matrix (CM), which categorizes results into True Positives (*TP*), False Positives (*FP*), True Negatives (*TN*), and False Negatives (*FN*). The metrics are computed as follows:

(1) Accuracy measures the overall correctness of the model and is defined as the proportion of correct predictions over the total number of predictions:

(14) $$\mathrm{Accuracy}=\frac{TP+TN}{TP+TN+FP+FN}$$

(2) Precision calculates the proportion of true positive predictions among all positive predictions made by the model:

(15) $$\mathrm{Precision}=\frac{TP}{TP+FP}$$

(3) Recall measures the proportion of true positives identified out of all actual positive samples:

(16) $$\mathrm{Recall}=\frac{TP}{TP+FN}$$

(4) F1-Score provides the harmonic mean of precision and recall, balancing the trade-off between them:

(17) $$F1\text{-}\mathrm{score}=2\times \frac{\mathrm{Precision}\times \mathrm{Recall}}{\mathrm{Precision}+\mathrm{Recall}}$$

### 3.2. Ablation Study

#### 3.2.1. Performance of MHA in Single-Label Prediction

To evaluate the effectiveness of MHA, we conducted experiments comparing the baseline model (without MHA) and the proposed MAL-Net (with MHA) across five subtypes: Qi-Yin Deficiency, Wind-Dampness, Blood Stasis, Liver-Wind, and Ni-Du. The results demonstrate that MAL-Net consistently outperforms the baseline model across most subtypes and evaluation metrics. Notably, accuracy and recall show significant improvements, which are critical for achieving accurate and balanced classification.

As presented in Table 2, the experimental results are based on five-fold cross-validation, ensuring stability and reproducibility. Given the minimal fluctuations across folds, the final performance metrics are reported as the average of all five folds. MAL-Net exhibits substantial performance improvements, particularly for Qi-Yin Deficiency and Blood Stasis. Specifically, MAL-Net achieves an accuracy of 0.98 and precision of 0.99 for Qi-Yin Deficiency, compared to the baseline’s accuracy of 0.85 and precision of 0.76. Similarly, for Blood Stasis, MAL-Net improves accuracy from 0.83 to 0.93. These results underscore the effectiveness of the MHA module in capturing complex and overlapping data patterns, particularly enhancing the identification of true positive cases in Qi-Yin Deficiency and Blood Stasis.

For subtypes such as Liver-Wind and Ni-Du, MAL-Net further demonstrates its robustness. While the baseline model achieves slightly higher accuracy for Liver-Wind (0.90 vs. 0.86), MAL-Net compensates with a higher precision of 0.93, highlighting its ability to reduce false positives. For Ni-Du, MAL-Net significantly enhances performance, yielding substantial improvements in both precision and recall and increasing the AUC from 0.88 to 0.96. These results underscore the effectiveness of the MHA mechanism in enhancing subtype classification by improving model sensitivity and precision, ultimately leading to better overall classification performance across diverse and challenging subtypes.

**Figure 5:**
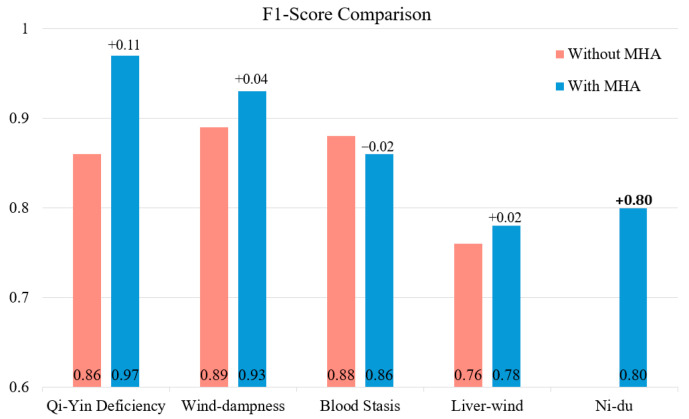
F1-score comparison: subtype classification performance with and without MHA.

Figure 5 illustrates the average F1-scores of the baseline model (without MHA) and MAL-Net (with MHA) across the five subtypes (Qi-Yin Deficiency, Wind-Dampness, Blood Stasis, Liver-Wind, and Ni-Du) based on five-fold cross-validation. The integration of MHA leads to substantial performance improvements, particularly for Qi-Yin Deficiency (+0.11, from 0.86 to 0.97) and Ni-Du (+0.80, from 0.00 to 0.80). Moderate gains are observed for Wind-Dampness (+0.04) and Liver-Wind (+0.02), while Blood Stasis shows a minor decline (−0.02). These results highlight the significant impact of MHA in enhancing classification performance, particularly for challenging subtypes, by improving the balance between precision and recall.

#### 3.2.2. Hyperparameter Optimization

During deep learning model training, performance gradually stabilizes as the number of iterations increases, with parameters converging toward optimal values. To determine these optimal parameters, extensive experiments were conducted exclusively on the training set, ensuring that model evaluation remained unbiased and robust. Performance was rigorously assessed using multiple evaluation metrics, and all hyperparameters were selected based on these training results, explicitly avoiding any reference to the testing dataset.

(1) Effect of learning rate

**Figure 6:**
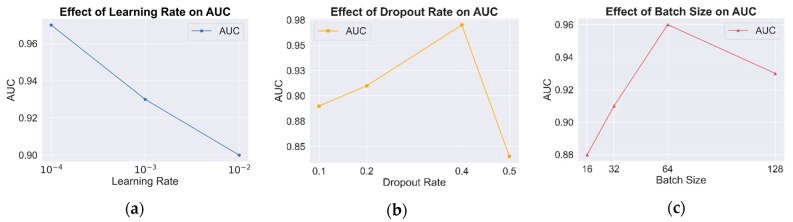
AUC comparison under different hyperparameter configurations. (**a**) Results of learning rate; (**b**) results of dropout rate; (**c**) results of batch size.

The learning rate plays a crucial role in minimizing the loss function, significantly impacting model convergence and overall performance. A high learning rate may lead to unstable training, while a low learning rate can result in prolonged convergence or suboptimal performance. To address this, ablation experiments were conducted to identify the optimal learning rate, as shown in Table 3 and Figure 6a. The results indicate that as the learning rate decreases, AUC gradually increases, reflecting improved classification performance. At a learning rate of 0.0001, the model achieves its highest AUC, indicating optimal performance. Therefore, a learning rate of 0.0001 is selected as the optimal setting.

(2) Effect of dropout rate

To evaluate the role of the dropout rate in mitigating overfitting and enhancing generalization, experiments were conducted by manually adjusting the dropout rate and analyzing its impact on model performance. The results in Figure 6b indicate that a moderate dropout rate effectively prevents overfitting while improving classification performance. However, when the dropout rate is too high (e.g., 0.5), excessive information loss occurs, leading to a decline in classification accuracy. The results confirm that the model achieves optimal classification performance at a dropout rate of 0.4.

(3) Effect of batch size

The batch size significantly affects both convergence speed and model generalization. Larger batch sizes improve computational efficiency but may lead to overfitting or unstable training, whereas smaller batch sizes typically result in smoother convergence at the cost of longer training times. To determine the optimal batch size, classification performance was evaluated across batch sizes of 16, 32, 64, and 128, as shown in Figure 6c. The results indicate that the model achieves optimal performance with a batch size of 64. Increasing the batch size to 128 causes premature convergence to local optima, reducing generalization performance. These findings suggest that a batch size of 64 is the most suitable configuration for this model.

### 3.3. Comparative Evaluation of Classification Models

To assess the effectiveness and classification performance of MAL-Net for IgAN subtype prediction, we conducted comprehensive comparative experiments. Six baseline models were implemented using the same dataset, including deep learning models (DNN [32], CNN [33], LSTM [22]) and traditional machine learning models (SVM [9], LR [11], RF [16]). These experiments aimed to evaluate various classification approaches in a multi-label classification setting and identify the most effective model.

To ensure fair comparisons, all input data underwent preprocessing to extract multi-dimensional features, and hyperparameters were fine-tuned through preliminary experiments. Each model was trained and evaluated on identical training and testing sets, maintaining consistency and reliability. Model performance was evaluated using classification accuracy, precision, recall, F1-score, and AUC, offering a comprehensive assessment of predictive capabilities. A paired sample *t*-test was conducted to measure performance differences, and the corresponding *p*-values were reported. A summary of the classification results is provided in Table 4.

As presented in Table 4, the results demonstrate the superior performance of MAL-Net, achieving the highest average F1-score of 0.885 with minimal performance fluctuations, followed by LSTM. In contrast, DNN and CNN exhibited weaker performance with higher variability.

**Figure 7:**
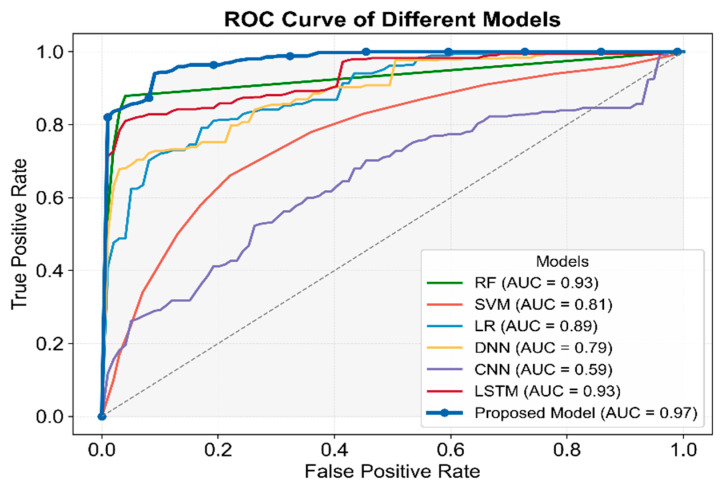
Comparative analysis of ROC and AUC for seven classifiers.

As shown in Figure 7, MAL-Net outperformed all models in AUC, achieving the highest value of 0.97, underscoring its strong classification ability. While LSTM and RF also performed well (AUC ≈ 0.93), CNN recorded the lowest AUC of 0.59. The *p*-values for all models were less than 0.05, confirming statistical significance. Among traditional machine learning models, RF exhibited the strongest performance, outperforming LR and SVM, with SVM trailing behind. Among deep learning models, LSTM emerged as the most competitive, whereas DNN and CNN lagged significantly. These findings reinforce that MAL-Net surpasses other classification methods, particularly due to its higher average AUC, reflecting robust predictive performance.

### 3.4. Performance Evaluation of Multi-Label Classification

This section evaluates the performance of the proposed model in multi-label classification tasks from two perspectives: overall multi-label classification and individual subtype classification, as analyzed using ROC curves.

**Figure 8:**
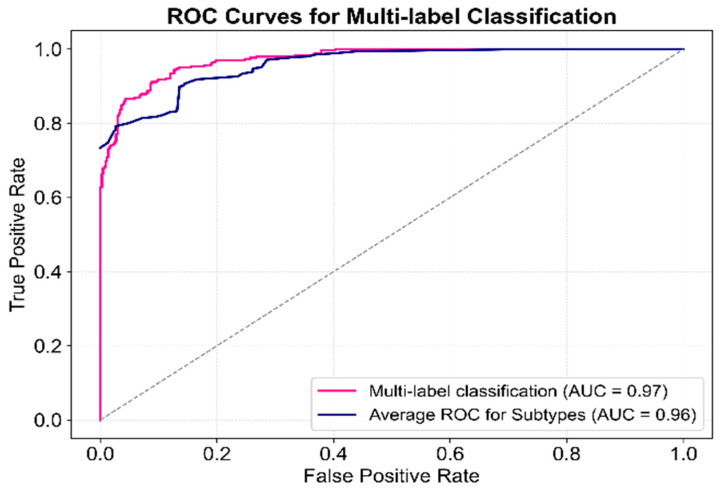
ROC curve for multi-label classification performance of the proposed model.

The multi-label classification results and average subtype classification performance are presented in Figure 8. The ROC curve for multi-label classification, computed by aggregating predictions across all subtypes, achieved an AUC of 0.97, demonstrating excellent overall classification performance. Additionally, the subtype-level ROC curves, calculated individually per subtype and then averaged, yielded an average AUC of 0.96, confirming robust performance across individual subtype predictions. The close proximity of the two curves suggests minimal impact from class imbalance, highlighting the model’s high stability, though minor variations persist. Furthermore, the rapid convergence of both curves toward the top-left corner emphasizes the model’s high sensitivity and specificity.

**Figure 9:**
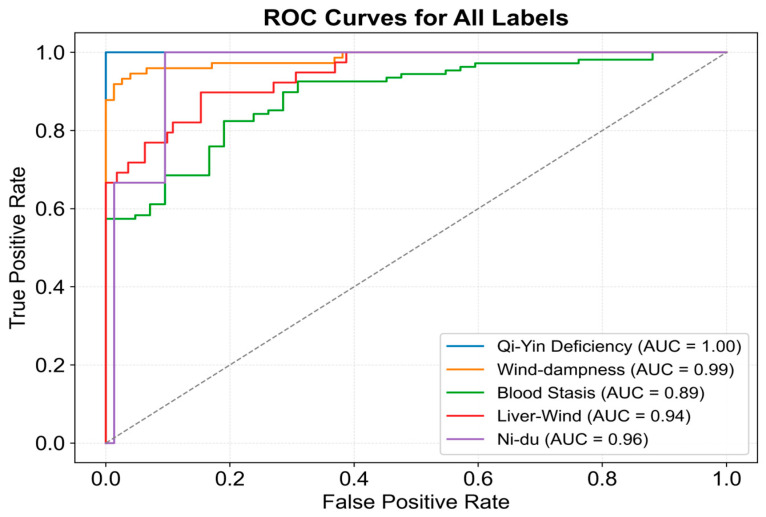
ROC curve for subtype classification of the proposed model.

The difference in AUC between the two curves suggests potential classification bias for certain subtypes. To investigate this, classification results for each subtype were visualized in Figure 9. The model exhibited high recognition ability for most subtypes; however, Blood Stasis displayed a slightly lower AUC, although its ROC curve maintained a smooth upward trend. In contrast, the Ni-Du subtype exhibited larger fluctuations, particularly at higher false positive rates, indicating instability in discrimination at certain thresholds. This instability may be due to the smaller sample size, which could limit feature extraction accuracy. The remaining subtypes demonstrated minimal fluctuations in their ROC curves, with consistently high AUC values, confirming the model’s ability to achieve high classification accuracy across most subtypes.

**Figure 10:**
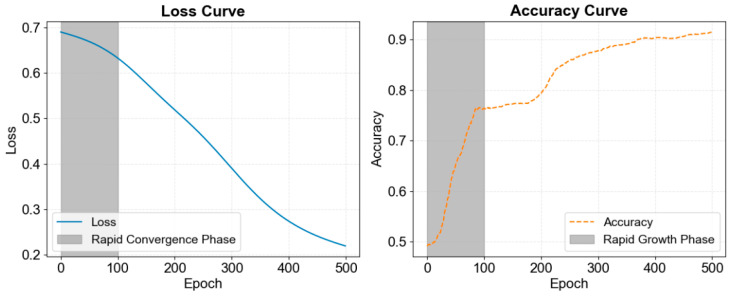
Loss curve and accuracy curve during training of MAL-Net.

The training loss and accuracy curves are shown in Figure 10. During training, the model progressively learned from the data, with the loss function value decreasing steadily. The loss converged rapidly around 100 epochs, dropping from approximately 0.7 to 0.2, indicating a significant reduction in prediction error. Simultaneously, accuracy increased progressively, rising from 0.5 to over 0.9. The accuracy curve displayed rapid growth around 100 epochs, suggesting that the model learned key features during this phase. After this point, the growth rate slowed, but around 200 epochs, another notable increase in accuracy was observed, likely due to further weight optimization and deeper feature learning. Eventually, accuracy stabilized, showcasing the model’s enhanced learning capacity. These results confirm the model’s strong generalization ability, resistance to overfitting, and capacity for high classification performance in complex multi-label tasks.

## 4. Discussion

**Figure 11:**
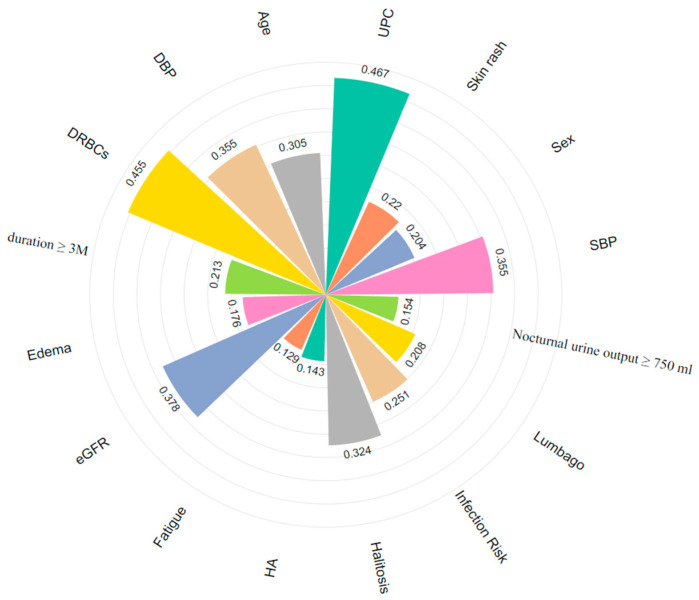
Key features for multi-label classification of IgAN (different colors correspond to different features). HA: Headache.

This study integrates multidimensional clinical data and enhances the model’s interpretability through feature importance analysis. We conducted importance scoring on disease factors influencing multi-label classification, selecting the top 16 features and visualizing them in Figure 11. This analysis identifies key influencing factors and provides intuitive support for clinical decision-making.

DRBCs, eGFR, and UPC, as traditional biomarkers, have been widely used in the diagnosis and prognosis assessment of IgAN [16]. Additionally, certain clinical symptoms provide supplementary value in subtype classification. For example, lumbago (0.208) and edema (0.176) may indicate renal inflammation activity [15]. Due to their ease of observation and prominent manifestations, they are significant in assessing disease severity. Including these features in diagnostic workflows can enhance patient evaluation. While ROC curves and overall AUC metrics effectively demonstrate MAL-Net’s classification capability, subtype-specific trends warrant further discussion. We observed significant variability in classification accuracy across IgAN subtypes, influenced primarily by clinical presentation and dataset characteristics. For example, the Qi-Yin Deficiency subtype, characterized by distinct clinical manifestations and a sufficient sample size, achieved high identification accuracy due to clearer patterns and distinguishable features. Conversely, the Ni-Du subtype, which represents a severe disease stage, commonly exhibits overlapping symptoms with other subtypes, making it more challenging to differentiate. Consequently, the model struggled to extract key differentiating features, resulting in lower classification performance for this subtype.

The significant performance improvements demonstrated by MAL-Net hold important clinical implications for IgAN subtype diagnosis. Enhanced prediction accuracy and robustness offered by MAL-Net can assist clinicians in earlier and more precise identification of IgAN subtypes, enabling targeted and personalized treatment strategies. Accurate subtype prediction may facilitate proactive intervention, improved disease management, and optimized therapeutic outcomes, directly benefiting patient care. Practically, integrating MAL-Net into clinical decision-support systems has the potential to streamline diagnostic workflows, reduce clinical workload, and enhance diagnostic consistency, thereby contributing to better clinical decision-making.

Ablation experiment results confirm that the memory network module effectively processes multidimensional clinical data. Dropout layers significantly enhance the robustness and generalization capabilities of MAL-Net. By randomly deactivating neurons during training, dropout mitigates overfitting and reduces model variance. Experimental results indicate that a moderate dropout rate (0.3–0.5) substantially improves model stability and generalization, whereas excessively high dropout rates negatively impact model capacity. These findings align with previous research [31]. Furthermore, our feature selection approach ensures that the model focuses on the most relevant clinical features, reducing redundancy and improving predictive accuracy. Separate experiments confirmed that careful feature selection notably enhances model efficiency and accuracy, highlighting its critical role alongside the LSTM and MHA modules. MAL-Net successfully differentiates all subtypes, benefiting from the Matt module, which addresses the challenge of subtype differentiation. Additionally, the MHA mechanism captures complex feature dependencies, improving the model’s sensitivity to key information and enhancing classification performance. Notably, the F1-score for the Ni-Du subtype increased by 0.8, validating the effectiveness of the MHA mechanism in handling class imbalance issues.

This study employs a multi-head self-attention (MHSA) mechanism, enabling the model to simultaneously attend to different feature interactions and dependencies within the same input set. Compared to single-head self-attention, MHSA divides input features into multiple subspaces, effectively capturing complex internal relationships. While cross-attention focuses on interactions between different modalities or feature sets, it is particularly beneficial in multimodal scenarios. Given that our IgAN subtype predictions rely on homogeneous clinical data, MHSA is the most appropriate choice. However, cross-attention may be explored in future studies involving multi-source or multimodal datasets.

To illustrate these advantages, we have included a comparative analysis of parameter counts and computational complexity for all studied models, as summarized in Table 5. As shown in Table 5, MAL-Net strikes a favorable balance between computational complexity and predictive performance. Although incorporating MHA increased parameter count from 8.3 K (baseline) to 14.6 K and computational load from 0.16 M to 0.49 M FLOPs, our model remains significantly more efficient than CNN (1.9 G FLOPs), DNN (500 M FLOPs), RF (50 M FLOPs), and SVM (5 M FLOPs). The computational efficiency and low parameter count make MAL-Net particularly suitable for resource-limited clinical environments and real-time diagnostic applications. Future research could further optimize computational efficiency using techniques such as pruning or low-rank approximations, enhancing the practical clinical applicability of the proposed model.

To further evaluate the efficiency of the proposed model, this study compared MAL-Net with mainstream classification models. Traditional machine learning (ML) methods have demonstrated effectiveness in early IgAN studies but require further evaluation in multi-label classification tasks [15,34]. Among them, Random Forest (RF) exhibited relatively balanced performance (AUC = 0.93) due to its ensemble learning advantages. However, these methods struggle with higher-dimensional features and an increased number of output labels, particularly in distinguishing positive and negative samples. Deep Neural Networks (DNN, AUC = 0.79) and Convolutional Neural Networks (CNN, AUC = 0.59) demonstrated weaker classification performance and lower robustness. While DNN has shown promise in some multi-label classification tasks, particularly in medical text classification [32], its deep structure often leads to overfitting and gradient vanishing issues. Similarly, CNN, which excels in image-based multi-label classification, is less effective on tabular and sequential data [33]. Future improvements could focus on optimizing model architectures and incorporating data augmentation techniques. The multidimensional nature of the data and the complexity of individual multi-label subtypes likely increase classification challenges for traditional methods, contributing to their performance decline. In summary, MAL-Net achieved the highest overall performance (AUC = 0.97), surpassing all other models across key evaluation metrics. Its superior classification capability highlights its potential as a highly effective and reliable tool for supporting clinical diagnosis in IgAN.

We acknowledge that IgAN datasets may inherently contain biases related to demographic factors such as age, sex, and ethnicity, as well as biases introduced by data collection methods. Although our dataset was carefully curated and validated, we did not conduct explicit fairness evaluations. Future studies should incorporate formal fairness analyses to quantify and mitigate potential biases, ensuring equitable model performance across diverse patient populations. Addressing these biases is crucial for achieving robust generalization and fair clinical applicability. While MAL-Net demonstrates strong feature extraction capabilities, further investigation into feature selection strategies informed by medical expertise is warranted. Expanding the feature set to include genomic and imaging data could further enhance classification accuracy and improve model interpretability.

## 5. Conclusions

This study presents MAL-Net, a novel multi-label deep learning framework for IgAN subtype classification, leveraging multidimensional clinical data, including sensor-derived variables such as laboratory indices and symptom tracking. By integrating LSTM networks and MHA mechanisms, MAL-Net effectively addresses the heterogeneity, class imbalance, and complex interdependencies inherent in clinical data. Experimental results demonstrate that MAL-Net significantly improves classification accuracy, outperforming both traditional machine learning and deep learning models. The ability to process sensor-based clinical data with high precision enhances MAL-Net’s clinical applicability, providing valuable support for early diagnosis, personalized treatment strategies, and prognosis evaluation. By combining real-time sensor data streams with advanced deep learning techniques, MAL-Net emerges as a promising tool for precision medicine, enabling more reliable disease stratification in complex conditions such as IgAN. Future work will explore the integration of additional sensor modalities, including imaging and genomic data, to further enhance model performance and broaden its applicability across diverse clinical settings.

## Figures and Tables

**Table 1:** Dataset characteristics and correlation analysis.

Characteristic	Training Set (*n* = 400)	Test Set	*p*-Value
		(*n* = 100)	
Age	38.88 ± 0.602	40.84 ± 1.067	0.084
Sex	Male (42.40%) Female (57.6%)	Male (44.80%) Female (55.20%)	0.640
SBP	125.15 ± 1.018	125.70 ± 1.639	0.577
DBP	77.52 ± 0.676	78.04 ± 1.241	0.629
UPC	1.53 ± 0.088	1.67 ± 0.225	0.973
DRBCs	0 (28.90%) 1 (12.20%) 2 (19.30%) 3 (32.80%) 4 (6.80%)	0 (28.70%) 1 (13.00%) 2 (18.30%) 3 (32.20%) 4 (7.80%)	0.993
eGFR	86.54 ± 1.638	83.36 ± 3.215	0.289
Lumbago	0 (63.30%) 1 (36.70%)	0 (59.50%) 1 (40.50%)	0.456
Fatigue/tiredness	0 (44.40%) 1 (55.60%)	0 (35.30%) 1 (64.70%)	0.082
Susceptible to colds	0 (76.70%) 1 (23.30%)	0 (81.00%) 1 (19.00%)	0.331
edema	0 (60.30%) 1 (39.70%)	0 (66.00%) 1 (44.00%)	0.456
Halitosis	0 (56.80%) 1 (43.20%)	0 (59.50%) 1 (40.50%)	0.615
Spontaneous/night sweats	0 (78.60%) 1 (21.40%)	0 (82.80%) 1 (17.20%)	0.325
Infection Risk	0 (66.10%) 1 (33.90%)	0 (68.10%) 1 (31.90%)	0.696
Skin rash	0 (63.00%) 1 (37.00%)	0 (59.50%) 1 (40.50%)	0.487
Muscle/body/joint soreness	0 (63.80%) 1 (36.20%)	0 (63.80%) 1 (36.20%)	0.995
Shortness of breath	0 (67.40%) 1 (32.60%)	0 (74.10%) 1 (25.90%)	0.171
Nocturnal urine output ≥ 750 mL)	0 (65.40%) 1 (34.60%)	0 (67.20%) 1 (32.80%)	0.710
Heat in palms and soles	0 (64.60%) 1 (35.40%)	0 (67.20%) 1 (32.80%)	0.600
Dry eyes	0 (65.10%) 1 (34.90%)	0 (67.20%) 1 32.80%)	0.673
Dry throat	0 (57.10%) 1 (42.90%)	0 (58.60%) 1 (41.40%)	0.772
Fixed lower back pain	0 (54.80%) 1 (45.20%)	0 (55.20%) 1 (44.80%)	0.941
duration ≥ 3 M	0 (64.60%) 1 (35.40%)	0 (65.50%) 1 (34.50%)	0.856
Skin purpura, petechiae/spider veins	0 (59.70%) 1 (40.30%)	0 (57.80%) 1 (42.20%)	0.710
Limb numbness	0 (62.80%) 1 (37.20%)	0 (57.80%) 1 (42.20%)	0.328
Dusky facial complexion	0 (55.00%) 1 (45.00%)	0 (57.80%) 1 (42.20%)	0.605
Irritability and anger	0 (82.20%) 1 (17.80%)	0 (83.60%) 1 (16.40%)	0.089
Headache	0 (80.60%) 1 (19.40%)	0 (82.80%) 1 (17.20%)	0.606
Blurry or darkened vision	0 (82.70%) 1 (17.30%)	0 (87.10%) 1 (12.90%)	0.262
Tremors, cramps	0 (78.30%) 1 (21.70%)	0 (81.00%) 1 (19.00%)	0.099
Anorexia, nausea	0 (96.60%) 1 (3.40%)	0 (94.00%) 1 (6.00%)	0.629
Dull complexion	0 (97.40%) 1 (2.60%)	0 (96.60%) 1 (3.40%)	0.620
Fear of cold	0 (96.40%) 1 (3.60%)	0 (94.00%) 1 (6.00%)	0.254

**Table 2:** Subtype classification results: with and without the MHA method.

Subtype	Method	Accuracy	Precision	Recall	AUC
Qi-Yin Deficiency	Baseline	0.85	0.76	0.99	0.98
	MAL-Net	0.98	0.99	0.95	1.00
Wind-dampness	Baseline	0.89	0.97	0.82	0.96
	MAL-Net	0.92	0.97	0.89	0.99
Blood Stasis	Baseline	0.83	0.80	0.99	0.80
	MAL-Net	0.93	0.78	0.96	0.89
Liver-wind	Baseline	0.90	0.85	0.68	0.94
	MAL-Net	0.86	0.93	0.68	0.94
Ni-du	Baseline	0.92	0.14	0.00	0.88
	MAL-Net	0.93	0.98	0.67	0.96

Baseline model: The baseline method employed for comparison is an LSTM model with the same overall network architecture, configuration, and hyperparameter settings as our proposed MAL-Net, but without the MHA module. MAL-Net: The proposed model, which integrates the LSTM with the MHA module.

**Table 3:** Evaluation metrics under different hyperparameter configurations.

Hyperparameter	Learning Rate			Dropout Rate				Bitch Size			
	0.01	0.001	0.0001	0.1	0.2	0.4	0.5	16	32	64	128
Accuracy (%)	81.2	86.8	91.9	88.3	90.6	90.7	89.3	80.5	85.6	91.6	89.2
Recall (%)	80.5	85.7	91.2	87.4	89.7	89.8	88.5	79.6	84.5	91.0	88.3
Precision (%)	82.1	87.2	92.0	88.9	91.2	91.3	89.4	81.2	86.0	92.0	89.7
F1-score (%)	81.3	86.4	91.6	88.1	90.4	90.5	88.9	80.4	85.2	91.5	89.0

**Table 4:** Comparison of experimental results for seven classification models.

Classification Model	Accuracy	Precision	Recall	F1-Score	AUC	*p*-Value
RF	0.892 ± 0.012	0.875 ± 0.015	0.850 ± 0.018	0.862 ± 0.014	0.915 ± 0.010	0.015
SVM	0.630 ± 0.020	0.635 ± 0.022	0.645 ± 0.025	0.620 ± 0.018	0.800 ± 0.015	0.036
LR	0.850 ± 0.015	0.780 ± 0.020	0.760 ± 0.022	0.770 ± 0.019	0.880 ± 0.012	0.029
DNN	0.620 ± 0.025	0.320 ± 0.030	0.450 ± 0.035	0.380 ± 0.028	0.780 ± 0.020	<0.01
CNN	0.670 ± 0.030	0.350 ± 0.035	0.430 ± 0.040	0.390 ± 0.032	0.575 ± 0.025	<0.001
LSTM	0.870 ± 0.011	0.700 ± 0.012	0.675 ± 0.015	0.690 ± 0.013	0.920 ± 0.008	0.043
**Proposed Model**	**0.905 ± 0.010**	**0.910 ± 0.010**	**0.855 ± 0.012**	**0.885 ± 0.011**	0.972 ± 0.006	

**Table 5:** Model comparison based on parameters and computational complexity.

Model	Parameters	FLOPs
RF	N/A	50 M
SVM	N/A	5 M
LR	0.03 K	0.05 M
CNN	15 M	1.9 G
DNN	2.5 M	500 M
LSTM (Baseline)	8.3 K	0.16 M
**Our Model**	14.6 K	0.49 M

## Data Availability

The raw data supporting the conclusions of this article will be made available by the authors on request.
